# Serum-Nutrient Starvation Induces Cell Death Mediated by Bax and Puma That Is Counteracted by p21 and Unmasked by Bcl-x_L_ Inhibition

**DOI:** 10.1371/journal.pone.0023577

**Published:** 2011-08-24

**Authors:** Frédérique Braun, Joséphine Bertin-Ciftci, Anne-Sophie Gallouet, Julie Millour, Philippe Juin

**Affiliations:** Institut de Recherche Thérapeutique de l'Université de Nantes, Centre de Recherche en Cancérologie Nantes Angers, Institut National de la Santé et de la Recherche Médicale UMR 892/Université de Nantes, Nantes, France; University of Illinois at Chicago, United States of America

## Abstract

The cyclin-dependent kinase inhibitor p21 (p21WAF1/Cip1) is a multifunctional protein known to promote cell cycle arrest and survival in response to p53-dependent and p53 independent stimuli. We herein investigated whether and how it might contribute to the survival of cancer cells that are in low-nutrient conditions during tumour growth, by culturing isogenic human colorectal cancer cell lines (HCT116) and breast cancer cell lines in a medium deprived in amino acids and serum. We show that such starvation enhances, independently from p53, the expression of p21 and that of the pro-apoptotic BH3-only protein Puma. Under these conditions, p21 prevents Puma and its downstream effector Bax from triggering the mitochondrial apoptotic pathway. This anti-apoptotic effect is exerted from the cytosol but it is unrelated to the ability of p21 to interfere with the effector caspase 3. The survival function of p21 is, however, overcome by RNA interference mediated Bcl-x_L_ depletion, or by the pharmacological inhibitor ABT-737. Thus, an insufficient supply in nutrients may not have an overt effect on cancer cell viability due to p21 induction, but it primes these cells to die, and sensitizes them to the deleterious effects of Bcl-x_L_ inhibitors regardless of their p53 status.

## Introduction

p21^cip1/Waf1^ (herein after named p21) is a member of the Cip/Kip family inhibitors of cell cycle progression that associates with the cyclin/CDK complexes and with PCNA, a processivity factor for replication polymerase, leading to the inhibition of CDK activities and DNA replication [1]. p21 is a p53 target gene and it is a relevant mediator of p53 induced cell cycle arrest in response to DNA damaging agents and/or oncogenic stress [1], [2]. Other studies have shown that p21 has additional functions as a differentiation inducer [3], [4] and as an inhibitor of apoptosis induced by DNA-damaging agents [5]. Given the importance of cell death induction in the clinical effects of chemotherapeutic drugs, this latter activity is likely to be critical, and to impede treatment efficiency [1], [6].

Although p21 may act as an apoptosis inducer in certain instances, results obtained in many models indicate it has an anti-apoptotic effect when cells are treated by genotoxic agents [7]–[10]. Moreover, some studies have reported that p21 promotes cell survival in response to antimetabolites, antimitotic and differentiating agents, and proteasome inhibitors [1], [11], [12]. This implies that p21 might play a role in the survival of cancer cells that goes beyond conditions of a p53 dependent response to acute genotoxic stress. By inference, overcoming its cytoprotective effects may represent a general, and critical, therapeutical issue. Most relevantly here, p21 was reported to promote, *in vitro*, the survival of embryonic fibroblasts grown in the absence of serum and treated by rapamycin [13]. Since the activity of the mammalian target of rapamycin (mTOR) is sensitive to serum and nutrient, this suggests that p21 might contribute to oncogenesis by playing a role in the adaptive response of cancer cells to the low-nutrient environment that typically characterizes the central region of solid tumors. Whether this is effective in human cancer cells, and what role p53 might play under these conditions, nevertheless needs to be elucidated.

Another key issue, partly unresolved, is the molecular mechanisms through which p21 promotes survival under each of the aforementioned conditions. p21 may indeed protect cells from apoptosis by diverse mechanisms. Firstly, these mechanisms may depend on p21 localization. Some data indicate that, under conditions of genotoxic stress, inhibition of the apoptotic response by p21 is linked to its ability to inhibit CDK activities in the nucleus [5], [7], [8]. In other cases, p21 was found to prevent apoptosis from the cytosol [4], [14], [15]. This process can be regulated by the ability of the survival Akt kinase to directly induce its phosphorylation and promote its retention the cytosol [16], [17]. Secondly, p21 may exert its effects at different levels of the apoptotic machinery. Apoptosis is a cell death process whose efficiency relies in great part on the activation of a cascade of cysteine active proteases, the so-called caspases. p21 was reported to bind to and to inhibit the effector caspase, caspase 3, and to do so from the cytosol [18]. However, cell death induced by caffeine on irradiated MCF7 breast cancer cells that do not express caspase 3, was linked to a decrease of the expression of p21 and, consistently, was counteracted by overexpressed p21 [10]. Therefore, the antiapoptotic function of p21 may not only involve its capacity to inhibit the caspase 3 and may act more upstream in the apoptotic machinery.

The Bcl-2 family of interacting proteins acts as major regulators of the mitochondrial apoptotic pathway, representing an integrating node towards which converge numerous death and survival signals in mammalian cells [19]. Anti-apoptotic Bcl-2 homologues preserve mitochondrial integrity by opposing the activity of multi-domain pro-apoptotic Bcl-2 family members Bax and Bak, which display sequence conservation throughout three Bcl-2 homology (BH) domains (BH1-3), and that of their upstream effectors, the BH3-only proteins (e.g. Bim, Puma, Bad…). This occurs essentially by physical interactions between anti- and pro-apoptotic members that involve engagement of the BH3 domain of the latter by a hydrophobic pocket formed at the surface of the former. This BH3-binding allows anti-apoptotic proteins to negatively control the activity of pro-apoptotic Bax itself, the essential actor of mitochondrial permeabilization and of the apoptotic response of mammalian cells to multiple stimuli [20]. These physical interactions play a key role in maintaining cell survival. More specifically, mechanistic investigations revealed that the interactions required to maintain survival are these that the anti-apoptotic proteins (such as Bcl-x_L_) engage with “activator” BH3-only proteins (such as Puma or Bim, which can directly activate Bax) and with multi-domain proteins themselves (such as Bax) [21], [22]. To the best of our knowledge, whether p21 might impact on some of these interactions to prevent cell death in some instances remains currently unknown. It should be noted, however, that induction of Puma upon DNA damage does not trigger apoptosis unless p21 expression is abolished, indicating that p21 can interfere with cell death induced downstream of Puma [7].

In this study, we investigated the role played by p21 in cell death induced by serum nutrient starvation. We show that starvation induces p21 independently from p53 and, that this mitigates the efficiency of a Puma/Bax dependent apoptotic signal also induced by starvation. We show, moreover that the anti-apoptotic function exerted by p21 in starved cells occurs upstream of mitochondrial permeabilization, at the level of Puma interactions with Bcl-x_L_. It is thus overcome by silencing of Bcl-x_L_ or by the inhibition of its BH3-binding activity using the ABT-737 compound, even in cells that carry a p53 mutation. Thus, starved cells might not die due to p21 induction, but they might be primed to die and sensitized to Bcl-x_L_ inhibitors such as ABT-737 regardless of their p53 status.

## Results

### p21 prevents efficient induction of cell death by serum/nutrient deprivation in HCT116 cells independently from p53

**Figure 1:**
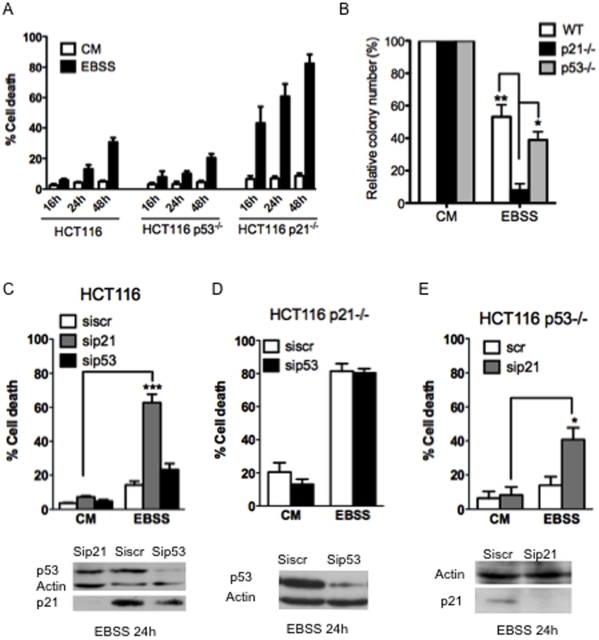
Role of p21 and p53 in cell death induced by serum nutrient starvation. (A) The indicated cells were placed in complete medium (CM) or in starved medium for 16 h, 24 h or 48 h (EBSS) and cell death was assayed by a trypan blue-staining. Data presented are mean ± SEM of three independent experiments. (B) The indicated cells were placed in complete medium (CM) or in starved medium during 24 h and then seeded at a low density for 2 weeks in complete medium before visualization of viable clones by crystal violet staining. (C, D and E) The indicated HCT116 cells were transfected with the indicated siRNA. 48 h later, cells were starved in EBSS medium for 24 h (EBSS) or not (CM). Western blot analysis was performed to confirm silencing and cell death was assayed by a trypan blue-staining. Data presented are mean ± SEM of three independent experiments.

We studied the potential role of p21 and of p53 in the response to serum-nutrient starvation using the wild type human colorectal cancer cells (HCT116 wt) and its isogenic counterparts deficient for either p21 (HCT116 p21−/−) or p53 (HCT116 p53−/−). For this purpose, HCT116 wt, HCT116 p53−/− and HCT116 p21−/− were cultured in minimal EBSS medium and cell death was measured. HCT116 cells deficient for p21 present a significant higher sensitivity to induction of cell death by starvation compared to the isogenic cells wt and p53−/− (Fig. 1A). p21 deficient cells undergo cell death after 16 h of starvation while 48 h of starvation are required to promote cell death in wild-type and p53 deficient cells. Colony formation assays were also performed to evaluate the long term response of the different cell lines to a period of serum-nutrient starvation. For that purpose, cells were cultured in complete medium or placed under starvation condition during 24 h and then seeded at a low density for 2 weeks in complete medium before visualization of viable clones (Fig. 1B). Whereas approximately half of wild-type HCT116 cells and p53 deficient cells formed colonies under these conditions, only one tenth of p21 deficient cells still formed colonies after the same treatment. Thus, p21 significantly delays cell death induced by serum-nutrient starvation.

As it was recently described that the hypersensitivity to apoptotic stimuli of HCT116 p21−/− cells could be due to enhanced p53 stability, and not to inactivation of p21 *sensu stricto* [23], we analyzed whether the enhanced sensitivity of HCT116 p21−/− cells to starvation induced cell death was a direct consequence of p21 absence. We thus directly down regulated its expression, using RNA interference, in HCT116 wt cells. Silencing of p21 was sufficient to sensitize HCTT116 wt cells to starvation induced cell death (Fig. 1C). In contrast, silencing of p53 had no effect on the viability of starved HCT116 wt cells and did not protect HCT116 p21−/− cells from starvation-induced death (Fig. 1 C–D, and Fig. S1A). Thus, p53 is dispensable for p21 sensitive induction of apoptosis by starvation. Knock down of p21 was efficient to sensitize to starvation induced cell death in HCT116 p53−/− cells (Fig. 1E and Fig. S1B). Of note, p53 expression was not affected by knock down of p21 by RNA interference in HCT116 wt cells, suggesting that cell death induced in p21 depleted cells is unlikely to result from increased p53 levels (Fig. 1C).

Taken together, these data indicate that the higher sensitivity of the HCT116 p21−/− cells to starvation is genuinely due to their lack of p21 expression, and that stabilisation of p53 is unlikely to play a role.

### Serum-nutrient starvation induced an apoptosis response mediated by Bax and Puma in p21−/− deficient cells

**Figure 2:**
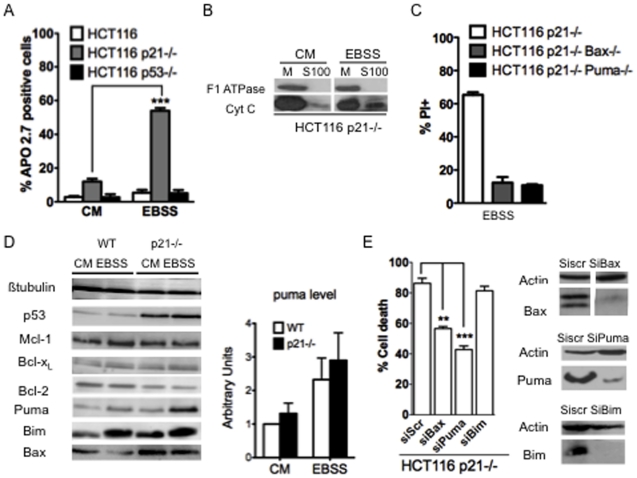
Puma and Bax as key actors of apoptosis induced by starvation in p21-negative HCT116 cells. (A) The indicated cells were placed in complete medium (CM) or in starved medium for 24 h (EBSS). Apoptosis was assayed by APO 2.7 staining using flow cytometry. Data are expressed as a percentage of the total population. (B) Mitochondria (M) and S100 (S100) lysates from HCT116 p21−/− cells, starved 16 h in EBSS medium (EBSS) or not (CM), were analysed by western blot analysis for F1ATPase and Cytochrome C (Cyt C). (C) Viability of the indicated cells placed under starvation during 24 h was analyzed by PI staining followed by flow cytometry. (D) The indicated cells were placed under complete medium (CM) or in starved medium (EBSS). After 24 h, protein extracts were prepared and the expression of the indicated protein was analysed by western blotting. The amount of Puma was evaluated in each condition by densitometric analysis from three independent experiments and normalized to the amount of Puma level measured in complete medium in wild-type cells (E) The p21 negative HCT116 cells were transfected with the indicated siRNA. 48 h later, cells were starved in EBSS medium for 24 h. Western blot analysis of Bax, Puma and Bim was performed and cell death was assayed by a trypan blue-staining. Data presented in (A), (C) and (E) are mean ± SEM of three independent experiments.

To test if the mitochondrial apoptotic pathway could account for starvation induced cell death in HCT116 p21−/−, we measured apoptosis levels in wild-type, p21−/− and p53−/− HCT116 cells placed 24 h in starved medium (EBSS) or in complete medium (CM) as a control. Apoptosis was assayed by quantification of the expression of the APO2.7 marker by flow cytometry. Under the conditions used, significant rates of apoptosis were only detected in starved HCT116 p21 −/− cells whereas signals detected in starved wt and p53−/− cells were very low and comparable to these measured in cells grown under control conditions (Fig. 2A). Consistent with the notion that starvation triggered apoptosis in p21 deficient cells, we found, after subcellular fractionation, that starved HCT116 p21−/− exhibited released cytochrome C in starved condition (Fig. 2B).

Since pro-apoptotic Bcl-2 family members act as major actors of mitochondrial permeabilization, we analyzed whether such proteins contribute to p21-sensitive cell death signals induced by starvation. In order to investigate a role for Bax in starvation-induced apoptosis, we investigated the response of HCT116 p21−/− Bax−/− to starvation (Fig. 2C) and we found that cell death was not induced by serum-nutrient starvation in such cells. These data are in agreement with previous data which showed that serum-starvation induced mainly apoptotic features upon starvation of Bax expressing HCT116 cells and not in Bax deficient HCT116 cells [24].

As we have shown that, in HCT116 p21−/− cells, the BH3-only protein Puma plays a key role as an upstream activator of Bax [21], we also analysed the response to starvation of double deficient p21-Puma- cells. These cells remained essentially viable upon starvation, while cells only deficient for p21 presented a high mortality under the same conditions. This result indicates that, in the absence of p21, starvation induced cell death is mediated by Bax and Puma (Fig. 2C).

Analysis of the expression of several Bcl-2 members in parental cells or in cells deficient for p21 showed that the expression of two BH3-only proteins, Puma and Bim, was increased under starvation, regardless of the p21 status. In particular, a 2–3 fold increase in Puma levels was induced by starvation, both in wt and p21−/− cells (P value>0,05, Fig. 2D right panel). Bcl-x_L_, Bcl-2, Mcl-1 and Bax levels remained essentially unchanged by starvation, whether or not p21 was expressed in HCT116 cells (Fig. 2D).

The induction of Bim under starvation prompted us to investigate whether this protein was involved, as Puma, in starvation induced cell death. To measure the contribution of Bim, and to confirm that of Bax and Puma in starvation-induced apoptosis in HCT116 p21−/−, their expression was silenced by RNA interference. Efficiency of the siRNAs used was confirmed by western blot (Fig. 2E). HCT116 p21−/− cells proved slightly sensitive to transfection with control siRNA itself (note the slight increase in cell death in Figure 1D compared to Figure 1A). We could nevertheless observe that the Puma siRNA did protect HCT116 p21−/− from cell death induced by starvation similarly to the Bax siRNA (Fig. 2E), consistent with results obtained with the HCT116 p21−/− Bax−/− and HCT116 p21−/− Puma−/− cell lines (Fig. 2C). In contrast, down regulation of Bim had no effect on cell viability of starved p21−/− cells (Fig. 2E).

These data indicate that Bax and Puma, but not Bim, are key players of apoptosis induced by starvation in p21 deficient cells.

### p21 survival function does not involve interactions with caspase 3

**Figure 3:**
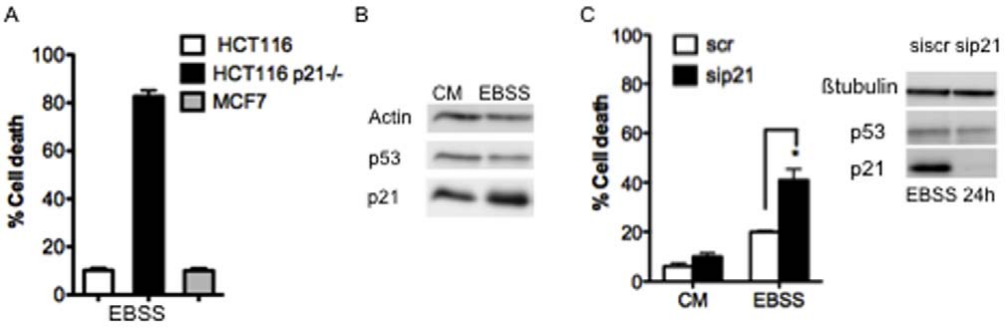
Survival function of p21 in the Caspase 3 deficient MCF7 cells. (A) The indicated cells were starved in EBSS medium for 24 h (EBSS) and cell death was assayed by a trypan blue-staining. (B) MCF7 cells were placed in complete medium (CM) or starved in EBSS medium (EBSS) for 24 h. Western blot analysis was performed to analyse p21 and p53 expression. (C) MCF7 cells were transfected with the indicated siRNA. 48 h later, cells were starved in EBSS medium for 24 h (EBSS) or not (CM). Western blot analysis of p21 and p53 was performed and cell death was assayed by a trypan blue-staining. Data presented in (A) and (C) are mean ± SEM of three independent experiments.

It was described that p21 could exert an anti-apoptotic function through its ability to physically interact with, and to inhibit, caspase 3 [18]. We thus reasoned that, if the cytoprotection exerted by p21 in starved cells relied solely on this interaction, then p21 might not protect cells that lack caspase 3 against starvation induced apoptosis. The breast cancer MCF7 cell line was used because these cells do not express caspase 3 due to a deletion mutation in exon 3 of their caspase-3 gene [25]. 24 hours of serum nutrient deprivation induced low rates of cell death in the MCF7 cells (Fig. 3A). p21 protein expression levels were indeed strongly increased under serum-nutrient starvation conditions in MCF7 cells while the expression of p53 remained stable (Fig. 3B). To determine if p21 was involved in the survival of starved MCF7 cells, RNA interference was used as before to efficiently silence p21 expression (Fig. 3C). As observed above in HCT116 cells, knock down of p21 did not detectably modify the expression of p53 in MCF7 cells. Measurement of cell viability showed that decreasing p21 expression sensitized MCF7 cells to induction of cell death by serum-nutrient starvation (Fig. 3C).

These data indicate that the induction of p21 by starvation, and the cytoprotection exerted by p21 extend to another cell line. They imply, moreover, that the anti-apoptotic activity of p21 under these conditions does not only rely on its interaction with caspase 3.

### p21 expression is enhanced by starvation independently from p53

**Figure 4:**
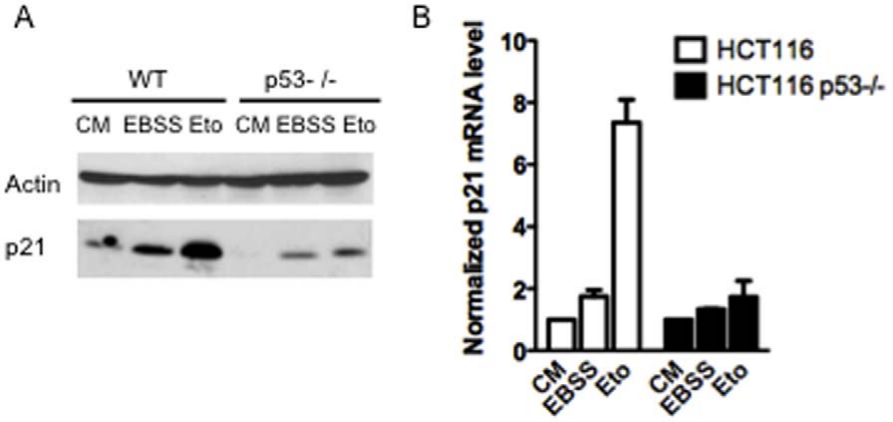
Serum-nutrient starvation enhancement of p21 protein levels. (A) The indicated cells were placed in complete medium (CM), treated 24 h with 50 µM of etoposide (Eto) or starved 24 h in EBSS medium (EBSS). Western analysis was performed to detect p21. (B) The indicated cells were placed in complete medium (CM), treated with 50 µM of etoposide (Eto) or starved in EBSS medium (EBSS) during 6 h for qPCR analysis. p21 mRNA level is expressed as relative values with two house-keeping genes and normalized to complete medium condition.

Under DNA damage, the protective effect of p21 is due to its transcriptional activation by p53 [1]. We thus investigated whether p21 expression was increased in response to starvation in HCT116 cells and whether p53 impacted on this process. The p21 protein expression level was strongly enhanced in wild-type HCT116 cells placed under serum-nutrient starvation conditions, even though this induction was rather weaker than that induced by etoposide, a genotoxic drug (Fig. 4A). Starvation induced increase of p21 protein level was also observed in p53 deficient HCT116 cells while, as expected, induction of p21 by etoposide in these cells was strongly affected (Fig. 4A). Consistently, p21 was still found induced in starved HCT116 wild-type cells in which p53 was silenced by RNA interference (Fig. 1C). To analyze whether enhancement of p21 expression occurred at a transcriptional level, RNAs were extracted from wild-type and p53 deficient cells cultured in control conditions (complete medium), placed under starvation or treated with 50 µM etoposide for 6 hours and Q-PCR analysis of p21 mRNA expression was performed (Fig. 4B). As expected, p21 mRNA was enhanced by etoposide in wild-type cells but not in p53 deficient cells. In contrast starvation had no major impact on p21 mRNA levels in either cell line, indicating that p21 induction by starvation may not occur at the level of transcription.

These results indicate that the induction of p21 in starved cells relies on a mechanism distinct from that involved in its induction upon DNA damage, in that it is neither p53 nor, more generally, transcription dependent.

### Cytoplasmic p21 exerts cytoprotection in starved cells

**Figure 5:**
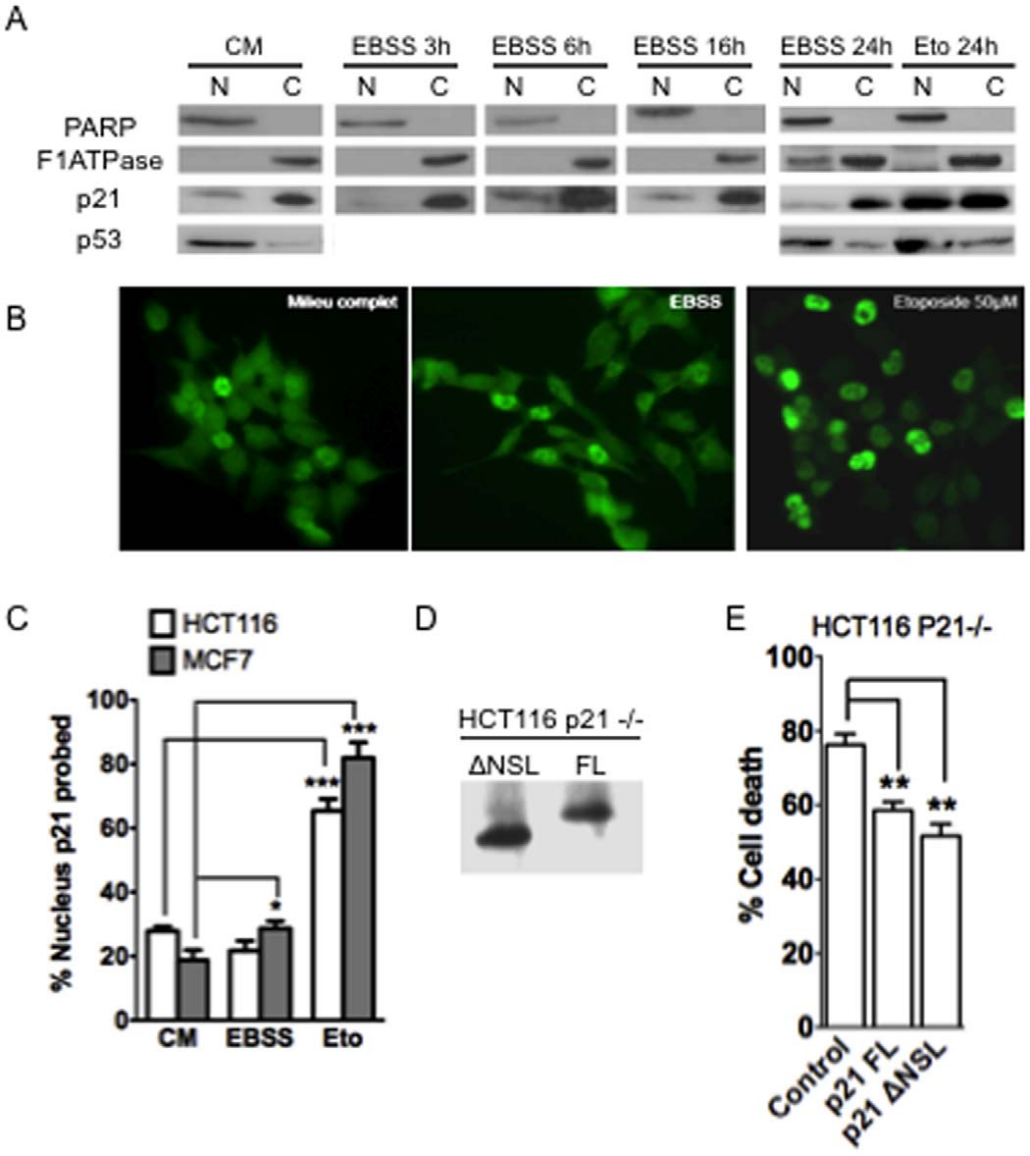
Impact of intracellular localization on the survival function of p21. (A) Nuclear versus cytoplasmic extracts prepared at the indicated times from HCT116 wt cells place under EBSS or not (CM), or treated with 50 µM of etoposide (Eto) for 24 h. Western analysis was performed for p21, p53 and F1ATPase and PARP. (B) Immunostaining of p21 in HCT116 wt cells placed under complete medium (CM), starved medium (EBSS) or treated with 50 µM of etoposide (Eto) for 24 h. Nucleus was stained with DAPI. (C) Quantification of immunostaining of p21 in HCT116 and MCF7 cells. The amount of cells containing a nuclear p21 localisation was expressed as a percentage of total cells detected with DAPI staining. (D) Western blot analysis of ectopic p21 in HCT116 p21−/− transfected with either pGFPires21FL expressing the full-length p21 protein (FL) or with pGFPires21ΔNSL (ΔNSL) expressing the p21 protein deleted from its C-terminal NSL domain. (E) HCT116 p21−/−transfected cells with the indicated vector were placed in EBSS medium and followed by time-lapse videomicroscopy. The vectors used allowed the expression of the GFP concomitant with p21, due to the presence of an IRES element. The percentage of transfected (ie GFP-expressing) cells exhibiting morphological features of cell death was assayed during 24 h. Data presented in (C) and (E) are mean ± SEM of three independent experiments.

The cellular functions of p21 depend upon the cytoplasmic or nuclear localization of the protein [4]. We thus analyzed the intracellular localization of p21 upon starvation by subcellular fractionation. As expected, a high level of p21 was found in the nuclear fraction of cells treated during 24 h with etoposide (Fig. 5A). In contrast, under starvation, p21 was found preferentially in the cytoplasmic fraction. This profile of expression was comparable to that observed in non-starved cells and it stayed essentially unchanged during the course of starvation (Fig. 5A). Analysis of the localisation of p53 indicated that it was mostly localized in the nucleus fraction in cells cultured in complete medium, under starvation or treated with etoposide (Fig. 5A).

To confirm these results, the localisation of p21 was determined by immunohistochemistry 24 h after starvation or genotoxic treatment in HCT116 and MCF7 cells (Fig. 5B and data not shown). Again, p21 was detected essentially in the cytoplasm of starved HCT116 cells and in that of HCT116 cells placed under control conditions (Fig. 5B). Quantitative analysis indicated that in either condition, p21 was present respectively in the nucleus of a limited, and comparable percentage of cells. In contrast, it was found preferentially localized in the nucleus of HCT116 cells treated with a genotoxic agent (Fig. 5C). Similarly, p21 localized in the cytosol of control and starved breast cancer MCF7 cells, while it preferentially localized in the nucleus of MCF7 treated with etoposide (Fig. 5C).

The cytosolic localization of p21 in starved cells suggests that p21 does not need to localize in the nucleus to exert its cytoprotective activity. To substantiate this, we deleted the nuclear localisation sequence of p21 located in the C-terminal region, leading to a truncated variant (ΔNLSp21), transduced it (or full length p21) in HCT116 p21−/− cells and induced starvation. Transfection of p21 deficient HCT116 cells with vectors containing the full-length p21 cDNA or the ΔNLSp21 mutant lead to expression of the corresponding p21 variant as confirmed by western blot (Fig. 5D). Analysis of the localisation of ectopic p21 by immunocytochemistry showed that full-length p21 protein was detected in both nucleus and cytoplasm while the truncated ΔNLSp21 protein remained in the cytoplasm as expected (data not shown). To discriminate successfully transfected cells from untransfected cells, we used a vector that allowed the expression of bicistronic mRNAs in which the gene encoding the green fluorescent protein (GFP) used as a marker was translated from the same mRNA as p21 (full length or lacking its NLS) due to the presence of an IRES sequence. This system allowed us to investigate, by time-lapse videomicroscopy, the ability of the different constructs to rescue HCT116 p21−/− cells from cell death induced by starvation, through quantification of the percentage of GFP positive cells that exhibited morphological features of cell death as a function of time under conditions of starvation (Fig. 5E). HCT116 p21−/− cells that expressed full-length p21 or the ΔNLSp21 mutant were both significantly less sensitive to starvation that the HCT116 p21− cells transfected with the empty vector.

Thus, the anti-apoptotic function of p21 under starvation does not require its nuclear localisation.

### The protective function of p21 is overcome by inhibition of Bcl-x_L_ in starved cells

**Figure 6:**
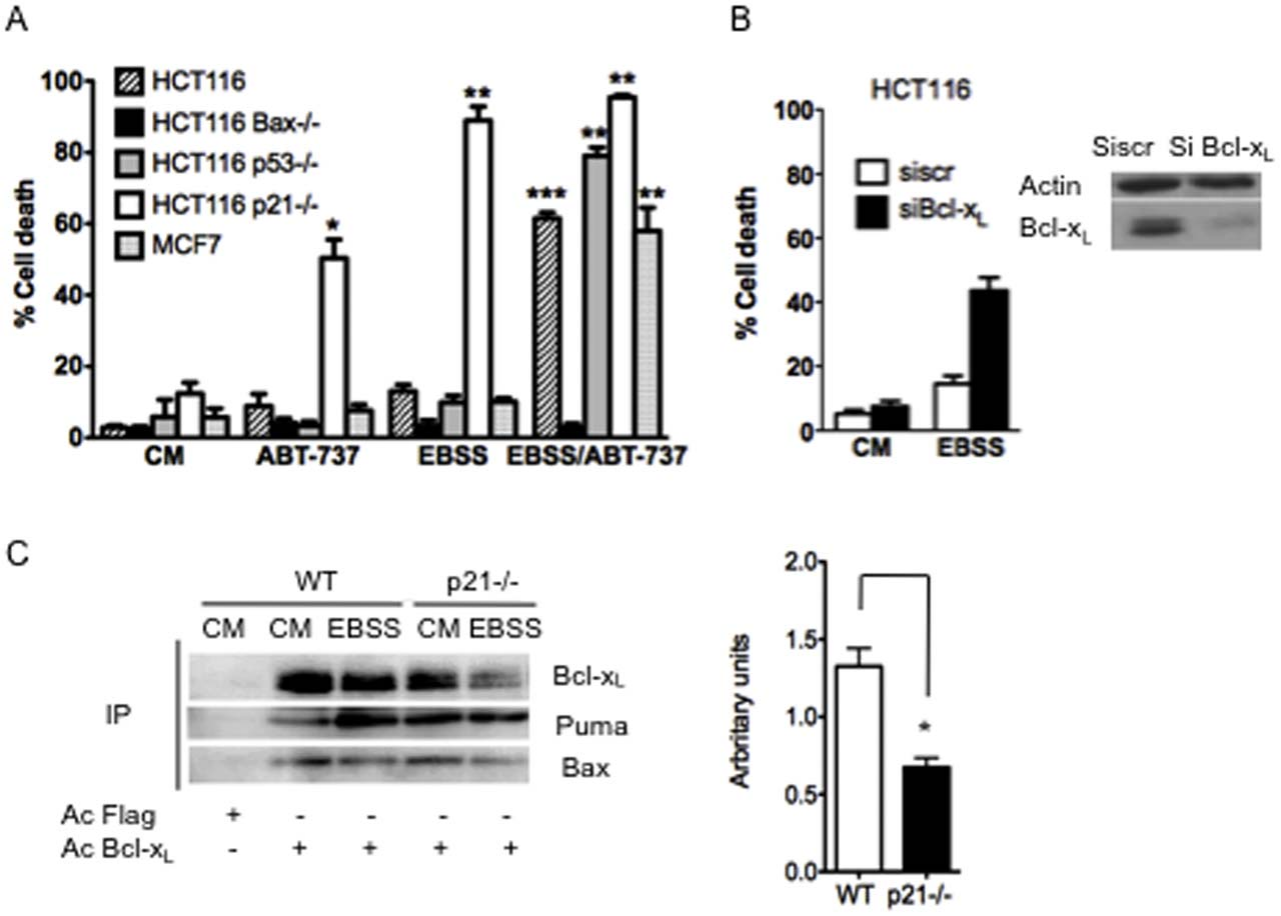
Sensitization to starvation induced cell death by down regulation or inhibition of Bcl-x_L_. (A) Inhibition of the BH3 domain of Bcl-2/Bcl-x_L_ sensitizes starved cells independently of p53. The indicated cells were placed in complete medium (CM) or starved in EBSS medium for 24 h (EBSS) and treated with 1 µM of ABT-737 or not and cell death determined by blue trypan assay (**, ***, significantly different from cells placed in complete medium). (B) HTC116 cells were transfected with the indicated siRNA. 48 h later, cells were starved in EBSS medium for 24 h (EBSS) or not (CM). Western analysis of Bcl-x_L_ was performed and cell death assayed by a trypan blue-staining. (C) Wild-type and p21−/− HCT116 cells were placed in complete medium (CM) or starved in EBSS medium for 24 h (EBSS). Expression of Bcl-x_L_ and Puma and Bax in cell lysates was analysed by western blot. Cells lysates were immunoprecipated (IP) with an anti-Bcl-x_L_ antibody or an anti-Flag antibody as a control, and the presence of Bcl-x_L_ and Puma and Bax in the immunoprecipitated fractions was analysed by immunoblotting. The amount of Puma that co-immunoprecipitated with Bcl-x_L_ (evaluated by densitometric analysis and normalized to the amount of immunoprecipitated Bcl-x_L_) in EBSS condition divided by the amount of Puma that co-immunoprecipitated with Bcl-x_L_ (evaluated by densitometric analysis and normalized to the amount of immunoprecipitated Bcl-x_L_) in complete medium condition are shown in wilt type and p21 deficient cells as indicated. Data presented in A, B and C are mean ± SEM of three independent experiments.

The data described above imply that p21 prevents cell death in starved cells at a level that occurs upstream of mitochondrial permeabilization, but downstream of Puma induction. Since anti-apoptotic Bcl-2 family members function in part by preventing Puma to directly activate Bax [19], we investigated whether inhibition of some of these proteins, by ABT-737, a potent inhibitor of the BH3 binding activities of Bcl-2 and Bcl-x_L_ [26], might overcome the protective function of p21. We tested the ability of ABT 737 to sensitize wild-type, p53 or Bax deficient HCT116 cells to serum nutrient starvation. Treatment of wild-type and p53 deficient HCT116 cell lines with 1 µM of ABT-737 significantly enhanced their cell death response to 24 h of starvation while viability of Bax deficient cells was not affected (Fig. 6A). MCF7 cells were also sensitised to ABT-737 when combined to serum nutrient starvation condition (Fig. 6A). Notably, and in contrast, ABT-737 barely affected the already high cell death rates induced by starvation in HCT116 p21−/− cells (Fig. 6A).

To confirm that at least one target of ABT-737 was involved in the effects reported above, we investigated whether depletion of Bcl-x_L_ phenocopied the effects of ABT-737 in starved cells. Bcl-x_L_ was silenced by RNA interference in HCT116 wild-type cells and the efficiency of the siRNAs used was checked by western blot (Fig. 6B). Knockdown of Bcl-x_L_ expression by RNA interference proved sufficient to sensitize wild-type HCT116 to starvation (Fig. 6B). Taken together, these data indicate that the BH3-binding activity of Bcl-x_L_ contributes to p21-mediated survival in starved cells.

We inferred from the assays above that p21 might impact on some of the interactions Bcl-x_L_ engages with its proapoptotic counterparts, and that changes in these interactions might account for the differential sensitivity of the wild type and p21−/− HCT116 cells in response to starvation. To test this hypothesis the intermolecular interactions between endogenous Bcl-x_L_, Puma and Bax were analysed by coimmunoprecipitation experiments in wild type and p21 deficient HCT116 cells placed in complete medium or under starvation. The amount of Puma bound to Bcl-x_L_ was increased upon starvation of wild-type cells but this did not seem to be the case in p21−/− cells (Fig. 6C). We quantified, from three independent experiments, Puma levels that coimmunoprecipitated with Bcl-x_L_ in wt or p21−/− cells that were starved or grown in control conditions. This confirmed that Puma bound to Bcl-x_L_ was significantly increased by starvation in cells expressing p21, and that this was not the case in p21−/− cells (Fig. 6D). Since Puma expression is induced by starvation regardless of p21 status, and since Bcl-x_L_ levels are similar in all conditions (Fig. 2D), this observation suggests that, in starved p21 expressing cells, the quantity of Puma molecules free of Bcl-x_L_ is lower than that in starved p21 deficient cells. Of note, the interaction between Bcl-x_L_ and Bax appeared not to be modified under starvation in either cell line (Fig. 6C).

These data indicate that starvation affects Bcl-x_L_ interactions with Puma differentially in wild-type and p21 deficient cells. Consistently, the anti-apoptotic function exerted by p21 in starved cells can be overcome by silencing of Bcl-x_L_ or by the inhibition of its BH3-binding activity, even in cells that lack p53 expression.

## Discussion

Under conditions that activate p53, the induction of p21 by p53 was shown to highly contribute to counteract apoptotic signals also induced downstream of p53 [1], [4], [5]. The work described here establishes that under conditions of nutrient deprivation, p21 protects cells from induction of apoptosis in a completely different setting. p21 may thus not only be an actor of the response of tumor cells to chemotherapy, but also contribute to their aberrant survival during tumor growth, even in the absence of treatment.

Recently, a possible caveat in the use of HCT116 p21−/− cells was reported, as their high sensitivity to two non genotoxic drugs, imatinib and gefitinib, was incriminated to the coincidentally high levels of p53 present in these cells rather than to p21 deficiency [23]. We show here that the direct silencing of p21 expression in wild type HCT116 and in another cell line suffices to sensitize these cells to starvation induced cell death, and its ectopic expression in HCT116 p21−/− cells promotes resistance against starvation induced cell death. These data clearly demonstrate that p21 protects cells against starvation-induced apoptosis.

One specificity of the cytoprotective function reported here for p21 is that it does not appear to counteract a p53 dependent death signal, and that it is thus susceptible to be functional even in tumor cells that carry p53 mutations. Indeed, the silencing of p21 similarly sensitized wild-type or p53−/− HCT116 cells to starvation. Moreover, the silencing of p53 did not modify the response of the HCT116 p21−/− cells to starvation.

Another feature that distinguishes the role played by p21 in starved cells from that it plays in DNA damaged cells is that p21 induction by starvation appears to be neither p53 dependent (as judged by the increase in p21 protein levels in starved p53 deficient cells) nor transcriptional (as judged by the lack of overt effect of starvation on p21 mRNA levels). Our observations recall that p21 was reported induced by serum released, independently of p53, not only at a transcriptional but also at a post-transcriptional level [27]. We did observe that serum withdrawal induced p21 but at a lower level than that induced by depletion of both serum and amino acids, indicating that the combined starvation is required for a full induction of p21 (Fig. S2). We speculate that p21 induction in response to serum-nutrient starvation may result from a stabilisation of the protein due to posttranslational modifications that most likely occur in the cytoplasm [16] [28]. Consistently, our data indicate that the anti apoptotic function of p21 in starved cells is associated with its cytoplasmic localisation. No modification of the localisation of p21 was observed in HCT116 and MCF7 cells placed under starvation and p21 remained mostly in the cytosol while as expected, etoposide almost induced a complete nuclear localisation of p21. Since the ectopic expression of a p21 protein deleted from its NSL domain was as capable as the full-length p21 protein to protect p21−/− HCT116 cells from apoptosis, the nuclear localisation of p21 appeared not required to counteract starvation-induced apoptosis. Several studies strongly suggest that p21 may have death-inhibitory functions in the cytoplasm independently of its ability to induce cell cycle arrest via inhibition of caspase 3 or kinase activities [14], [15], [17], [18]. However, our data indicate that the survival function of p21 in response to starvation may not solely deal with its ability to inhibit the pro-caspase 3, as silencing of p21 promoted cell death in starved MCF7 cells that do not express caspase 3. In agreement with this, when transfected with an antisense p21 oligodeoxynucleotide MCF7 cells undergo apoptosis associated to the attenuation of p21 levels [29].

Our data argue that, in starved cells, p21 counteracts a mitochondrial apoptotic pathway that relies on Bax and on its upstream effector Puma to be efficient. Triggering of this pathway by starvation most likely relies on the induction of Puma expression under these conditions. Q-PCR data indicate that starvation induces Puma mRNA levels and that it does so even in p53 deficient cells (data not shown). Consistently, the increased of Puma protein level was also observed in p53 −/− cells (Fig. S3) indicating that the mechanism responsible for Puma induction might be p53 independent. Thus, the difference of p53 level between wt and p21−/− cells may not interfere with Puma induction by starvation. Consistently, serum-nutrient depletion was found to increase Puma at a similar level in wt and p21−/− cells, indicating that p21 does not impact on Puma induction, and that p21 prevents cell death despite Puma induction. Under serum starvation, Puma was also described increased in various cancer lines that are mutant or null for p53, and it was reported to play a major role in the apoptotic response under these conditions [30]. The two transcriptions factors SP1 and p73 may be involved in this transcriptional activation of PUMA [30].

Our data suggest that Puma might form under starvation different complexes with Bcl-x_L_ in wild-type and p21 deficient HCT116 cells and we believe that this accounts, at least in part, for the difference of sensitivity between these cells. Thus, a complete mechanistic understanding of the cytoprotective effect of p21 requires to understand how it regulates Puma Bcl-x_L_ interactions. Since p21 does not appear to impact on the expression of other Bcl-2 family members such as Mcl-1 that might indirectly impact on these interactions, we speculate that post-transcriptional modifications of either partner may be involved. Phosphorylation of Bcl-x_L_ at the ser62 residue was reported to influence its interaction with Bax in certain conditions [31]. Yet, we were not able to detect under starvation Bcl-x_L_ phosphorylated at the ser62 in the HCT116 cell lines (data not shown). The phosphorylation status of Bax, Puma, Bcl-x_L_ and p21, their implications in starvation-induced apoptosis and the kinase(s) potentially involved need to be further characterized. One interesting candidate pathway is the one that relies on the c-Jun N-terminal Kinase (JNK). This kinase is activated by environmental stresses including serum nutrient starvation, and it was described to play a role in the regulation of the function of some Bcl-2 proteins [32], [33]. JNK1 also plays a role in the transcriptional regulation of Puma [34]. Moreover, JNK1 phosphorylates p21 leading to the inhibition of its ubiquitination and its stabilisation in p53 null K562 cells under oxidative stresses [35]. Conversely, p21 might mitigate this pathway by interfering with ASK1 [15].

Regardless of the exact mechanism involved in the cytoprotective effect exerted by p21 in starved cells, our data imply that they converge towards anti-apoptotic Bcl-2 proteins, and that the inhibition of these proteins should overcome this effect. Consistent with this, silencing of Bcl-x_L_ was sufficient to sensitize HCT116 cells to starvation. Similar results were obtained using ABT-737, which indicates that the inhibitory effect of Bcl-x_L_ involves its BH3 binding activity. Strikingly, serum-nutrient withdrawal combined with the ABT 737 compound induced cell death in wild-type or p53−/− HCT116 cell lines and in breast cancer cell lines containing a wild-type p53 or mutant p53 protein (such as the p53 mutant (R280K) MDA-MB 231 and (R249S) BT549 cells (data not shown).

Thus, p21 expressing, starved cells are primed to die independently of their p53 status resulting in a high sensitivity to ABT-737. Notably, p21 protein is overexpressed in various human cancers and this upregulation correlates positively with aggressive tumours (for list of references see Table 1 in Abbas T *et al*, 2009) [28]. More specifically, cytoplasmic expression of p21, which is common in human malignancies, was shown to be of bad prognosis in breast cancer [36], [37]. Our data imply that this may result from contextual cues, and reflect the existence of cells that resist to the deleterious effects of starvation. They imply, moreover that the use of compounds such as ABT-737 may overcome the survival, and promote the destruction, of tumor cells that express cytoplasmic p21.

## Materials and Methods

### Cell lines and cell culture

Human colorectal cell lines derived from HCT116 (p21−/−, p53−/−, p21−/−Puma−/− and p21−/−Bax−/−) were kindly provided by Dr B. Vogelstein (The John Hopkins Kimmel Cancer Center, Baltimore, MD). The MCF7 (ATCC-HTB-22) cell line was obtained from ATCC. All cell lines were incubated at 37°C and humidified 5% CO_2_-95% air in growth media are supplemented with 2 mM L-glutamine, 100 U/mL penicillin, 100 µg/mL streptomycin and 10% fetal calf serum (FCS), unless otherwise stated. HCT116 cells were grown in McCoy's 5A and MCF7 in RPM1-1640 supplemented with 0,023 units/ml bovine insulin. To induce serum nutrient starvation, cells were washed twice in PBS and incubated in Earle's Balanced Salt Solution (EBSS) supplemented with 0.1% BSA and 0.1 g/L MgCl_2_. When specified, etoposide were used at 50 µM. ABT-737 was synthesized as previously described and used at 1 µM (24).

### Plasmids, siRNAs and transfection

The full–length p21 gene and the p21 gene deleted from its NLS domain (24 amino acids at the C-terminal end) were cloned into the pGFPires vector (Clontech) after amplification by PCR using the sense primer: 5′- ATGTCAGAACCGGCTGGGGAT-3′ and the antisense primer 5′-TTAGGGCTTCCTCTTGGAGAAG -3′ or 5′- TTATCGACCCTGAGACTGCCT-3′. The plasmids, named pGFPires21FL and pGFPires21ΔNSL respectively, were sequenced. pGFPires, pGFPires21FL and pGFPires21ΔNSL were transfected using Lipofectamine 2000 (Invitrogen) into HCT116 p21−/− cells and the expression of the ectopic p21 proteins was checked by western blot analysis. The siRNAs were purchased for siScramble (SC37007), sip21 (SC29427) from Santa Cruz, sip53 (AMS1331) from Applied Biosystem, siBax (60730232) from IDT DNA, siPuma (4380) and siBcl-x_L_ (3458) from Dharmacon and siBim (6461) from Cell Signaling. Cells were transfected using the Hiperfect transfection reagent (QIAGEN) according to manufacturer's instructions and were incubated with nucleotides for 48 h before subsequent experiments.

### RNA extraction and quantitative real-time PCR

Total RNAs were extracted with RNAeasy mini kit (Qiagen). The quality of the RNAs was assessed by analysis the 28S∶18S rRNA ratio using the RNA 6000 Nano Assay kit and the Agilent 2100 bioanalyser (Agilent Biotechnologies). One microgram of RNA was retro-transcribed using Supers cript transcriptase (Superscript II, Invitrogen). Following foward and reverse primers designed with Amplifix software were used: p21 (5′-CAGCATGACAGATTTCTACCAC -3′ and 5′-GAGACTAAGGCAGAAGATGTAGAG -3′), rplpO (5′-GATGACCAGCCCAAAGGAGA -3′ and 5′- GTGATGTGCAGCTGATCAAGACT-3′), ß2microglobulin (5′-GGCATCTTCAAACCTCCATGATG -3′ and 5′- TTCACCCCCACTGAAAAAGATGA -3′). Real time PCR was carried out using the Brilliant Sybr Green QPCR core reagent kit (Stratagene) and the thermocycler of the MX-4000 multiplex Quantitative PCR system (Stratagene). Quantitative normalization of cDNA in each sample was performed using rplpo and β_2_-microglobulin mRNAs as internal control. Relative quantification was carried out using the ΔΔCt method.

### Immunoblot analysis and antibodies

Total proteins were extracted in 1% NP-40, 0.5% sodium-deoxycholate, 0.1% SDS supplemented with protease inhibitor Mini® from Roche. Nuclear extracts were obtained by subcellular fractionation using the subcellular protein fractionation kit (Thermo Scientific Pierce) according to the manufacturer's instructions. Mitochondria and S100 fractions were obtained by subcellular fractionation. The cells were scraped using a Teflon scraper and centrifuged at 1,000 *g* for 5 min at 4°C. The cell pellets were washed twice with ice-cold phosphate-buffered saline (PBS) and resuspended in volume-per-volume cell extraction buffer (CEB) (250 mM sucrose, 50 mM HEPES [pH 7.4], 50 mM KCl, 2 mM MgCl2, 1 mM dithiothreitol, 10 M cytochalasin B, 1 mM EGTA, and 1 tablet protease inhibitor). Cells were allowed to swell for 30 min on ice prior to their homogenization with 50 strokes in an ice-cold 2-ml glass Dounce homogenizer. Unbroken cells and nuclei were pelleted by centrifugation at 750 *g* for 10 min at 4°C. Mitochondria were collected from the resulting supernatants by centrifugation at 13,000 *g* for 15 min at 4°C. Mitochondrial pellets were resuspended in volume-per-volume in CEB buffer. The S100 fraction was obtained by ultracentrifugation of the resulting supernatants at 100,000 *g* for 30 min at 4°C. The integrity of the nuclear, mitochondria and S100 extracts was checked by the analysis of the expression by immunoblot of the nuclear PARP protein and the mitochondrial F1ATPse protein. Protein concentrations were determined by the bicinchoninic acid (BCA) method. Protein extracts were separated by SDS-PAGE, transferred onto a PVDF membrane (Millipore) and revealed with a chemiluminescence kit (Millipore). For signal detection the membranes were incubated overnight with antibodies to p21 (556431, BD-pharmigen), p53 (554294, BD-pharmingen), actin (MAB1501R, Millipore), ß-tubulin (T0198, Sigma), Bax (A3533, Dako), Bcl-x_L_ (1018-1, Epitomics), Bim (AB170003, Millipore), F1ATPase (A21350, Molecular Probe), Flag (F1804, Sigma), Mcl-1 (sc819, Santa Cruz) PARP (AM30, Calbiochem) and Puma (PC686, Calbiochem). HRP-conjugated secondary antibodies were from Bio-rad.

### Cellular assays

For immunocytochemichal assays, cells were grown on gelatin-coated cover-slips, and placed under complete medium or serum-nutrient depletion during 16 h. Cells were then fixed in 4% paraformaldehyde for 30 min, permeabilized with 0.1% SDS for 10 min, blocked with 5% BSA in PBS for 15 min, then incubated with the primary antibody rabbit polyclonal anti-p21 (BD pharmigen) overnight at 4°C in 1% BSA in PBS and then with the secondary Alexa-Fluor 568 nm antibody (Molecular probes) for 1 h in 1% BSA in PBS at 37°C. Cells were mounted in ProLong Gold antifade reagent with DAPI (Invitrogen) and observed under a using a Zeiss Axiovert 200-M inverted microscope (Carl Zeiss, Le Pecq, France). Coimmunoprecipitation assays were performed as previously described using CHAPS lysates [21]. Cell viability was determined by blue trypan staining or by PI staining using flow cytometry. In these assay, analysis were performed on cells that had reached 70–80% confluence. “BD ApoAlert APO 2.7-PE” (BD Biosciences) was used to determine the percentage of apoptotic cells according to the manufacturer's instructions Cells were analyzed on a FACScalibur (BD Biosciences) using Cell Quest Pro software. A minimum of 10 000 events were acquired for each condition. The debris were excluded from the analysis according to their FSC/SSC properties. For colony formation assays, cells were cultured in complete medium or placed under starvation condition during 24 h. Then, 500 cells were seeded in each well of a 6-well plate for 2 weeks in complete medium, after cells were stained with 0.5% crystal violet and 20% methanol.

### Time-lapse analysis

Time-lapse video-microscopy experiments were performed using a Leica DMI 6000B. Dishes were placed inside an Incubator chamber to a CO_2_ controller that maintained the environmental CO_2_ concentration at 5% for the duration of filming. Digital pictures were acquired using MetaMorph v.7.5 software (MDS, Foster City, CA) and saved every 10 min over 24 h using a CollSNAP HQ2 camera. The series of photographs were displayed as continuous time-lapse movies for analyses.

### Data analysis

Data were from at least three independent experiments. Statistical analysis of data was performed using one tail Student's t-test on GraphPad Prism. Error bars represent standard error of means (SEM). The following symbols are used: *, **, *** that correspond to a P value inferior to 0.05, 0.01 or 0.001 respectively.

## Supporting Information

Figure S1 **Role of p21 and p53 in cell death induced by serum nutrient starvation.** (A and B) The indicated HCT116 cells were transfected with the indicated siRNA. 48 h later, cells were starved in EBSS medium for 24 h (EBSS) or not (CM). Viability of the indicated cells placed under starvation during 24 h was analyzed by PI staining and flow cytometry. Data are mean ± SEM of three independent experiments.
(PDF)

Figure S2 **Enhancement of p21 protein level in serum withdraw medium.** HCT116 wild-type cells were placed in complete medium (CM), in serum withdrawn medium (SW) or starved 24 h in EBSS medium (EBSS). Western blot analysis was performed to detect p21 expression.
(PDF)

Figure S3 **Enhancement of Puma protein level in p53−/− HCT116 cells.** HCT116 p53−/− cells were placed in complete medium (CM) or starved 24 h in EBSS medium (EBSS). Western blot analysis was performed to detect Puma expression.
(PDF)
