# Supplementary figures and images for: Serum-Nutrient Starvation Induces Cell Death Mediated by Bax and Puma That Is Counteracted by p21 and Unmasked by Bcl-xL Inhibition
Source: PLoS One. 2011 Aug 24;6(8):e23577. doi: 10.1371/journal.pone.0023577 (PMC3160893; doi:10.1371/journal.pone.0023577)

Figure S1

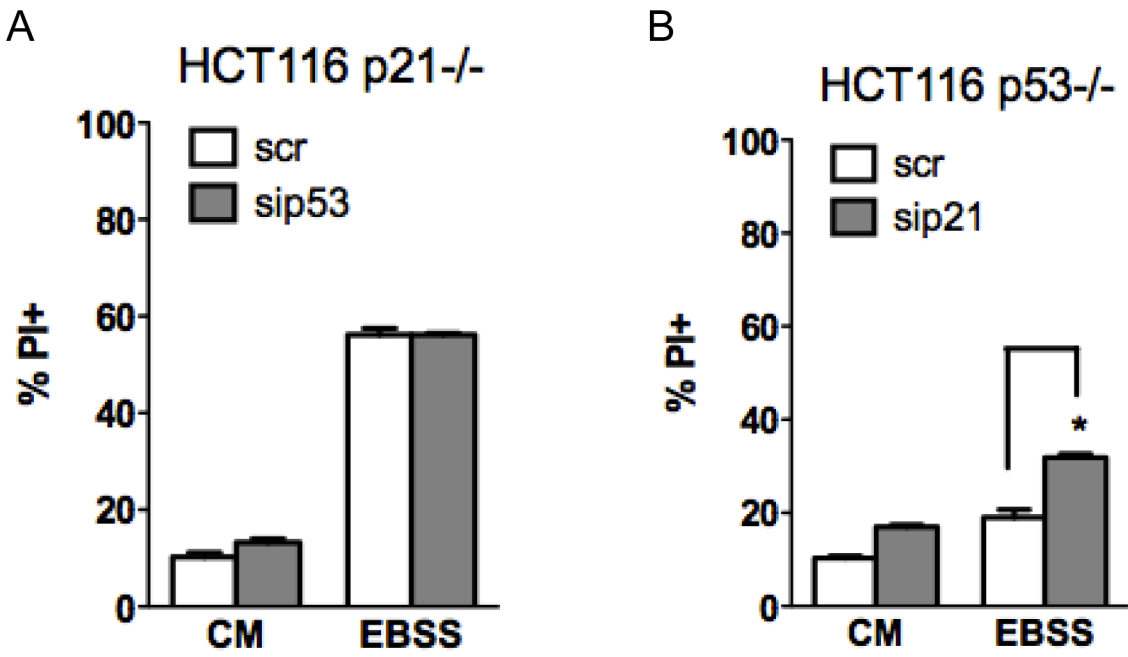

Supplement: Figure S1 — Role of p21 and p53 in cell death induced by serum nutrient starvation. (A and B) The indicated HCT116 cells were transfected with the indicated siRNA. 48 h later, cells were starved in EBSS medium for 24 h (EBSS) or not (CM). Viability of the indicated cells placed under starvation during 24 h was analyzed by PI staining and flow cytometry. Data are mean ± SEM of three independent experiments. (PDF) [file pone.0023577.s001.pdf]

Figure S2

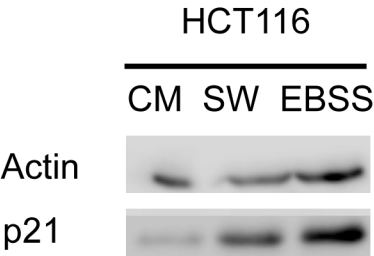

Supplement: Figure S2 — Enhancement of p21 protein level in serum withdraw medium. HCT116 wild-type cells were placed in complete medium (CM), in serum withdrawn medium (SW) or starved 24 h in EBSS medium (EBSS). Western blot analysis was performed to detect p21 expression. (PDF) [file pone.0023577.s002.pdf]

Figure S3

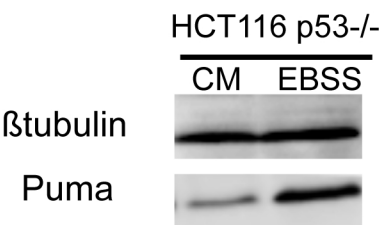

Supplement: Figure S3 — Enhancement of Puma protein level in p53−/− HCT116 cells. HCT116 p53−/− cells were placed in complete medium (CM) or starved 24 h in EBSS medium (EBSS). Western blot analysis was performed to detect Puma expression. (PDF) [file pone.0023577.s003.pdf]
